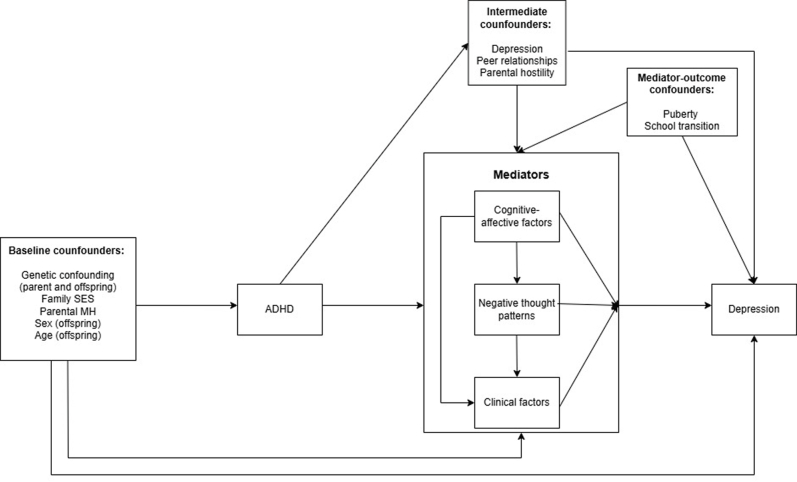# Study Preregistration: Clinical and Cognitive Mediators Underlying Subsequent Depression in Individuals With Attention-Deficit/Hyperactivity Disorder: A Developmental Approach

**DOI:** 10.1016/j.jaac.2025.03.023

**Published:** 2025-11

**Authors:** Eglė Padaigaitė-Gulbinienė, Gemma Hammerton, Jon Heron, Olga Eyre, Giorgia Michelini, Alexandra Wilson-Newman, Clara S. Garavini, Thalia C. Eley, Anita Thapar, Lucy Riglin

**Affiliations:** a Cardiff University, Cardiff, United Kingdom; b University of Bristol, Bristol, United Kingdom; c Queen Mary University of London, London, United Kingdom; d Wolfson Centre for Young People’s Mental Health Youth Advisory Group, Cardiff, United Kingdom; e McPin Young People’s Advisory Group, London, United Kingdom; f King's College London, London, United Kingdom; g South London and Maudsley Hospital, London, United Kingdom

## Abstract

Individuals with attention-deficit/hyperactivity disorder (ADHD) are about 5.5 times more likely to develop depression,^1^ and this comorbidity is associated with greater impairment than either disorder alone. Although there is evidence that ADHD may play a causal role in the development of depression,^2^^,^^3^ the underlying mechanisms remain poorly understood. Several clinical and cognitive mechanisms have been proposed: (1) clinical antecedents of depression, such as irritability and anxiety, often observed in individuals with ^4^^,^^5^; (2) cognitive–affective functions (response inhibition, working memory, sustained attention, and emotion recognition) impaired in individuals with ADHD and, to a lesser extent, in depressed individuals^6^^,^^7^; or (3) negative thought patterns underlying vulnerability to depression also observed in individuals with ADHD (external locus of control and negative cognitive styles).^8^ Nevertheless, few longitudinal studies have tested these as potential mediators between ADHD and subsequent depression. Existing studies are primarily cross-sectional, limited by small sample sizes, and have not examined developmental stage-specific effects. Therefore, we will explore the mediating role of clinical, cognitive–affective, and negative thought patterns, and whether their role varies by developmental stage and sex. We will examine all mediators simultaneously, the relative contribution of 3 categories of mediators, and the associations between ADHD and each hypothesized mediator/factor. We hypothesize the following: (1) ADHD will be more strongly associated with irritability and emotion recognition in childhood than in adolescence and young adulthood; (2) the association between ADHD and anxiety will be consistent across development; and (3) ADHD will be more strongly associated with response inhibition, working memory, sustained attention, external locus of control, and negative cognitive style in adolescence and young adulthood compared to childhood.

## Study Synopsis

### Introduction Summary

Individuals with attention-deficit/hyperactivity disorder (ADHD) are about 5.5 times more likely to develop depression,^1^ and this comorbidity is associated with greater impairment than either disorder alone. Although there is evidence that ADHD may play a causal role in the development of depression,^2^^,^^3^ the underlying mechanisms remain poorly understood. Several clinical and cognitive mechanisms have been proposed: (1) clinical antecedents of depression, such as irritability and anxiety, often observed in individuals with ADHD^4^^,^^5^; (2) cognitive–affective functions (response inhibition, working memory, sustained attention, and emotion recognition) impaired in individuals with ADHD and, to a lesser extent, in depressed individuals^6^^,^^7^; or (3) negative thought patterns underlying vulnerability to depression also observed in individuals with ADHD (external locus of control and negative cognitive styles).^8^ Nevertheless, few longitudinal studies have tested these as potential mediators between ADHD and subsequent depression. Existing studies are primarily cross-sectional, limited by small sample sizes, and have not examined developmental stage-specific effects. Therefore, we will explore the mediating role of clinical, cognitive–affective, and negative thought patterns, and whether their role varies by developmental stage and sex. We will examine all mediators simultaneously, the relative contribution of 3 categories of mediators, and the associations between ADHD and each hypothesized mediator/factor. We hypothesize the following: (1) ADHD will be more strongly associated with irritability and emotion recognition in childhood than in adolescence and young adulthood; (2) the association between ADHD and anxiety will be consistent across development; and (3) ADHD will be more strongly associated with response inhibition, working memory, sustained attention, external locus of control, and negative cognitive style in adolescence and young adulthood compared to childhood.

### Method Summary

Analyses will be performed in the primary sample (the Avon Longitudinal Study of Parents and Children [ALSPAC]; total N = 14,541) and replicated in the Twins Early Development Study (TEDS; total N = 16,810 twins). Ethical approvals for both studies were obtained from the ALSPAC Law and Ethics and Local Research Ethics and The King’s College London Ethics Committees.

In both cohorts, ADHD will be assessed using the hyperactivity/inattention subscale of the Strengths and Difficulties Questionnaire (SDQ), whereas depression will be assessed using the short Mood and Feelings Questionnaire (sMFQ). Irritability will be assessed using 3 items from the Oppositional Defiant Disorder (ODD) section of the Development and Well-Being Assessment (DAWBA) in ALSPAC and 1 item from the SDQ conduct subscale in TEDS. Anxiety will be assessed using the generalized anxiety subscale from DAWBA and the Screen for Adult Anxiety Related Disorders (SCAARED) in ALSPAC, and the Anxiety-Related Behaviours Questionnaire (ARBQ) and the Generalised Anxiety Disorder Dimensional Scale (GAD-D) in TEDS. Cognitive–affective functions will be examined in ALSPAC only. Emotion recognition will be assessed using the Diagnostic Analysis of Non-Verbal Accuracy (DANVA) and Emotion Recognition (ERT) tasks, while response inhibition - the Stop-Signal Task (SST). Working memory will be assessed using the Counting Span (CST) and N-Back (NBT) tasks. Sustained attention will be assessed using Sky Search Dual Task (SSDT) and Sustained Attention to Response Task (SART). Locus of control will be assessed using children’s Nowicki–Strickland Internal External Control Scale (CNSIE), whereas negative cognitive styles will be assessed using the Cognitive Styles Questionnaire Short Form (CSQ-SF).

**Figure 1:**
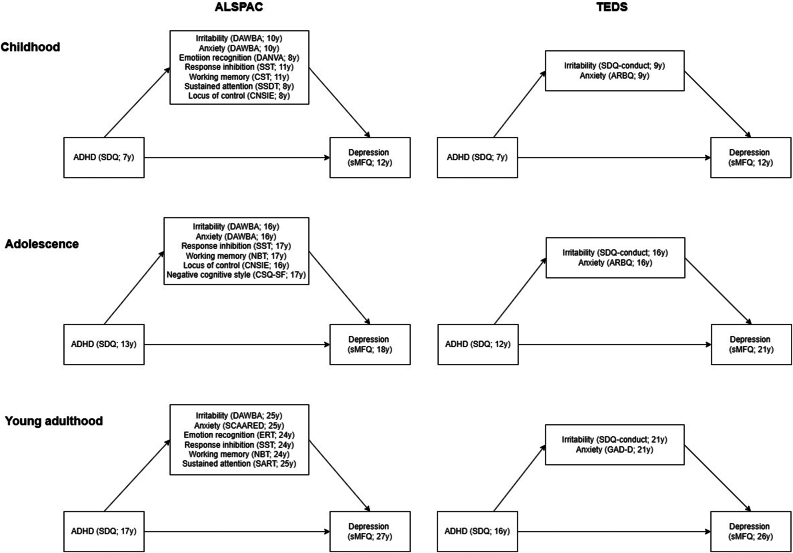
Time of Assessments for Attention-Deficit/Hyperactivity Disorder, Hypothesised Mediators, and Depressive Symptoms in Childhood, Adolescence, and Young Adulthood in Both Cohorts ***Note:****ALSPAC = Avon Longitudinal Study of Parents and Children; ARBQ = Anxiety-Related Behaviours Questionnaire; CNSIE = Children’s Nowicki–Strickland Internal External Control Scale; CSQ-SF = Cognitive Styles Questionnaire Short Form; CST = Counting Span Task; DANVA = Diagnostic Analysis of Non-Verbal Accuracy; DAWBA = Development and Well-Being Assessment; ERT = Emotion Recognition Task; GAD-10 = Generalised Anxiety Disorder Assessment; NBT = N-Back Task; SART = Sustained Attention to Response Task; SCAARED = Screen for Adult Anxiety Related Disorders; SDQ = Strengths and Difficulties Questionnaire; sMFQ = short Mood and Feelings Questionnaire; SSDT = Sky Search Dual Task; SST = Stop-Signal Task; TEDS = Twins Early Development Study.*

Mediation will be assessed using a counterfactual approach, which assumes no unmeasured confounding and allows the incorporation of exposure–mediator interactions.^9^ We will estimate pure natural direct effects (PNDE), total natural indirect effects (TNIE), and proportion mediated. First, the total proportion mediated will be estimated by including all mediators (Figure 1). Then, the effects of individual categories of mediators (ie, clinical, cognitive–affective, and negative thought patterns) will be examined. We will also examine whether potential mechanisms may vary by developmental stage or sex. A directed acyclic graph (DAG) will inform the choice of which variables to include in a model to adjust for potential confounders. Missing data will be imputed based on the missing data patterns and predictors of missingness.

### Significance Summary

Emerging evidence suggests that ADHD may have a causal impact on depression,^2^^,^^3^ but little is known about the mechanisms that could explain why many individuals with ADHD develop consequent depression. This study will identify clinical and cognitive mechanisms potentially underlying the development of depression in children and young people with ADHD, and whether the role of these mechanisms varies across developmental periods or sex. Previous research suggests that standard psychological interventions for depression may be less effective in children and young people with ADHD.^10^ Therefore, findings from this study have the potential to pinpoint those who are most at risk and to help identify targets for depression interventions tailored for individuals with ADHD.

## CRediT authorship contribution statement

**Eglė Padaigaitė-Gulbinienė:** Writing – review & editing, Writing – original draft, Conceptualization, Methodology. **Gemma Hammerton:** Writing – review & editing, Methodology, Supervision. **Jon Heron:** Writing – review & editing, Methodology, Supervision. **Olga Eyre:** Writing – review & editing, Methodology. **Giorgia Michelini:** Writing – review & editing, Methodology. **Alexandra Wilson-Newman:** Writing – review & editing, Methodology. **Clara S. Garavini:** Writing – review & editing, Methodology. **Thalia C. Eley:** Writing – review & editing, Methodology. **Anita Thapar:** Writing – review & editing, Methodology, Conceptualization. **Lucy Riglin:** Writing – review & editing, Supervision, Methodology, Funding acquisition, Conceptualization.

## Figures and Tables

**Supplementary Figure S1:**
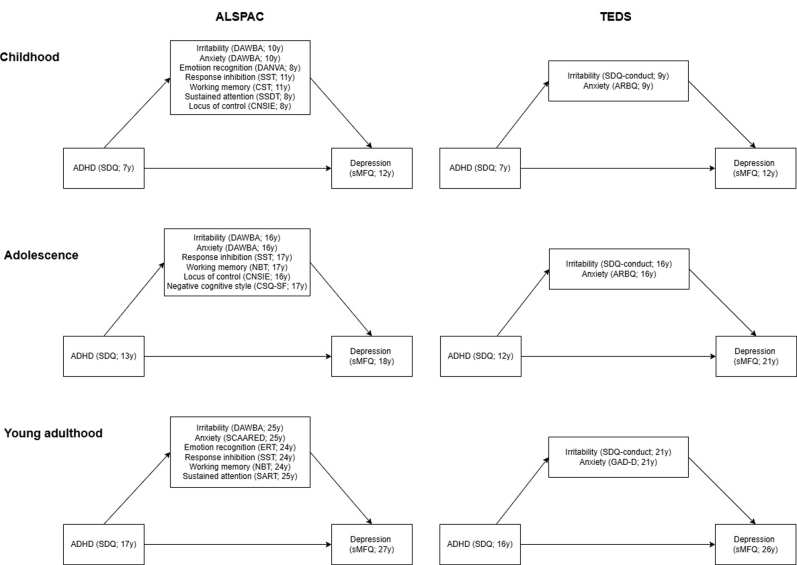


**Supplementary Figure S2:**