# Supplementary material for: Study Preregistration: Clinical and Cognitive Mediators Underlying Subsequent Depression in Individuals With Attention-Deficit/Hyperactivity Disorder: A Developmental Approach
Source: J Am Acad Child Adolesc Psychiatry. 2025 Nov;64(11):1329–31. doi: 10.1016/j.jaac.2025.03.023 (PMC13187293; doi:10.1016/j.jaac.2025.03.023)
Supplement: Supplementary Material [file mmc1.docx]

**Registered Report: Clinical and cognitive mediators underlying subsequent depression in individuals with ADHD: a developmental approach**

**Introduction**

Attention deficit hyperactivity disorder (ADHD) is characterised by impulsivity, inattention and hyperactivity.^1,2^ Although usually first diagnosed in childhood, ADHD symptoms often persist into adulthood, with an estimated 7.6% prevalence in childhood, 5.6% in adolescence, and 3.1% in adulthood.^1,2^ ADHD is often comorbid with psychiatric conditions such as substance use disorder (SUD), mood, anxiety, and personality disorders, of which depression is one of the most common.^3^ Individuals with ADHD are about 5.5 times more likely to develop depression compared to those undiagnosed, with an estimated prevalence of 18% experiencing depression by age 18, increasing to 44% by age 30.^4–6^

ADHD with comorbid depression has been previously linked with even greater functional impairment than either disorder alone, characterised by a higher risk of suicidal symptoms, chronic depression, recurrent hospitalisations, and poor treatment response in adult life.^7^ Emerging evidence suggests that ADHD may have a causal impact on depression.^8^ Nevertheless, little is known about the mechanisms that could explain why many individuals with ADHD develop subsequent depression. Identification of mechanisms that may elucidate the onset of depression in children and young people with ADHD could potentially pinpoint those most at risk and guide depression interventions for individuals with ADHD. In addition, previous research suggests possible developmental differences in depression intervention effectiveness for young people with ADHD. Although some psychological interventions for depression, such as cognitive behavioural therapy (CBT), can improve emotional symptoms in adults diagnosed with ADHD, evidence suggests that they may not be effective in children with ADHD.^9,10^ Therefore, it is crucial to examine clinical and cognitive mechanisms potentially underlying the associations between ADHD and subsequent depression and whether they vary across development.

Several clinical and cognitive-affective mechanisms have been proposed to potentially underlie the development of depression in individuals with ADHD. Irritability and anxiety have been identified as key clinical antecedents of depression^11^ that are also often observed in individuals with ADHD.^12,13^ Irritability is especially prominent in children with ADHD, while associations between ADHD and anxiety increase after puberty.^13,14^ Previous work has found that childhood irritability explained 42% of the association between childhood ADHD and depression in childhood/adolescence.^15^ Some studies observed that controlling for anxiety considerably attenuated the association between ADHD and depression in childhood^4^, while in adolescence/adulthood, results were inconsistent.^6,16^

Cognitive-affective domains commonly affected in ADHD include executive functions, such as response inhibition, working memory and sustained attention.^17,18^ Executive functions have also been associated, to a lesser degree, with depression.^19^ For example, working memory has been proposed as a crucial component in rumination,^20^ suggesting that these could be potential mediators of the associations between ADHD and subsequent depression.^21^ However, this research has been conducted on depression in adults, leading to the call for these associations with depression to be examined at younger ages. Research testing these as potential mediators between ADHD and depression has been limited to cross-sectional studies, which have provided some evidence of mediation for executive functions in adulthood, although they did not examine the role of sustained attention specifically.^22^

Another cognitive-affective domain which has been found to be associated with both ADHD and depression is emotion recognition. Previous research shows that compared to healthy controls, individuals with ADHD tend to demonstrate impaired emotion recognition.^23^ Although impairment was observed across development, emotion recognition was most severely impaired in children and adolescents and tended to improve with age.^24^ Similarly, depressed individuals tend to demonstrate impairments in facial emotion recognition and exhibit difficulties recognising anger, fear, disgust, surprise and happiness, but not sadness.^25^ To our knowledge, the mediating role of emotion recognition has not been tested previously.

Finally, cognitive^26^ and hopelessness theories of depression^27^ suggest that beliefs about self and the surrounding world are key cognitive factors underlying vulnerability to depression. Therefore, negative thought processes, such as external locus of control (LoC) (i.e. a tendency to think that positive and negative life events are caused by external factors outside one's control, such as fate, luck, or others) or negative cognitive styles (i.e. a tendency to attribute adverse life events to internal, global, and stable causes and belief that adverse events imply one is flawed or deficient) could be other potential routes to depression for those with ADHD. These are less well studied within the context of ADHD, although there is some evidence that individuals with ADHD tend to exhibit more external locus of control and are more likely to show maladaptive attributional styles for negative events (i.e. more internal, global and stable attributions).^28^ In cross-sectional studies, external locus of control mediated associations between ADHD and depression in older, but not younger children,^29^ while dysfunctional attitudes and cognitive-behavioural avoidance jointly mediated the ADHD association with depressive symptoms in male adults.^30^

Overall, few longitudinal studies have examined the potential clinical and cognitive-affective mechanisms underlying the associations between ADHD and subsequent depression. Available studies were primarily cross-sectional, limited by small sample sizes and did not examine whether these associations are developmental stage-specific. To address these limitations, we will aim to examine whether clinical (i.e. irritability and anxiety), cognitive-affective (i.e. response inhibition, working memory, sustained attention, and emotion recognition), and negative thought patterns (i.e. external locus of control and negative cognitive style) factors represent important mechanisms linking ADHD and subsequent depression and whether these vary across development (i.e. childhood, adolescence, and young adulthood) and sex. We will examine all mediators simultaneously, the relative contribution of 3 mediator categories (i.e. clinical, cognitive-affective, and negative thought patterns), and the ADHD associations with every factor/hypothesised mediator (e.g. emotion recognition). In particular, we hypothesise that:

**H_1_:** ADHD will be more strongly associated with irritability and emotion recognition in childhood than in adolescence and young adulthood.

**H_2_:** The association between ADHD and anxiety will be consistent across development.

**H_3_:** ADHD will be more strongly associated with response inhibition, working memory, sustained attention, external locus of control and negative cognitive style in adolescence and young adulthood compared to childhood.

Given possible sex differences in risk factors for depression,^31^ secondary analyses will examine whether mechanisms may vary by sex.

**Methods**

Analyses will be performed in the primary sample (i.e. the Avon Longitudinal Study of Parents and Children; ALSPAC) and replicated in the secondary/replication sample (i.e. the Twins Early Development Study; TEDS). Clinical mechanisms (i.e. irritability and anxiety) will be examined in both cohorts. However, cognitive-affective mechanisms are unavailable in the replication sample; therefore, they will only be examined in the primary sample.

**Samples**

**The Avon Longitudinal Study of Parents and Children (ALSPAC).** ALSPAC is an ongoing multi-generational longitudinal birth cohort comprised of mothers and their children born in South-West England between April 1st, 1991, and December 31st, 1992, who reside in the Avon area, UK.^32,33^ Pregnant women resident in Avon, UK, with expected delivery dates between 1st April 1991 and 31st December 1992, were invited to participate in the study. Of the initial 14,541 pregnancies, there was a total of 14,676 foetuses, resulting in 14,062 live births and 13,988 children alive at 1 year of age. When the oldest children were approximately 7 years of age, an attempt was made to bolster the initial sample with eligible cases who had failed to join the study. Therefore, the total sample size for analyses using any data collected after age seven is 15,447 pregnancies, resulting in 15,658 foetuses and 14,901 children alive at 1 year of age (for a more detailed description of the sample, see **Supplement 1**, available online). Data were collected from parents and their children during pregnancy and follow-ups through questionnaires, hospital and medical records, and clinic visits. ALSPAC is considered broadly representative of the UK population, although families participating in ALSPAC were more likely to be white and more affluent than families residing in the whole UK.^33^ The firstborn will be selected in twin pregnancies. Ethical approval for the study was obtained from the ALSPAC Law and Ethics and Local Research Ethics Committees. Informed consent for the use of data collected via questionnaires and clinics was obtained from participants following the recommendations of the ALSPAC Ethics and Law Committee at the time. Information on data available can be searched using a data dictionary and variable search tool: <http://www.bristol.ac.UK/alspac/researchers/our-data/>.

**The Twins Early Development Study (TEDS).** TEDS is an ongoing longitudinal birth cohort of twins born between 1994 and 1996 in England and Wales.^34^ The original sample comprised 16,810 families, of which 13,694 provided information at the children’s mean age of 18 months at the first assessment. The TEDS sample is primarily comprised of individuals of white ethnicity and is considered representative of families with young children in the mid-1990s in England and Wales.^35^ Both twins will be included in the analyses, controlling for the family structure of the data. The King’s College London Ethics Committee has provided ethics approval for TEDS. All available cohort data can be found on their website: <https://www.teds.ac.uk/datadictionary/home.htm>

**Measures**

**Table 1** describes the measures that will be used in this study.

**Attention deficit hyperactivity disorder (ADHD).** In both cohorts, ADHD symptoms will be assessed using parent-rated hyperactivity/inattention subscale of the Strengths and Difficulties Questionnaire (SDQ).^36^ In ALSPAC, ADHD symptoms assessed at ages 7, 13, and 17 will be used. In TEDS – at ages 7, 12, and 16.

**Depression.** Self-reported depressive symptoms will be assessed using the short Mood and Feelings Questionnaire (sMFQ)^37^ in both cohorts. In ALSPAC, depressive symptoms will be assessed at ages 12, 18, and 27, while in TEDS – at ages 12, 21, and 26.

**Irritability.** In ALSPAC, irritability will be assessed using 3 items from the Oppositional Defiant Disorder (ODD) section of the Development and Well-Being Assessment (DAWBA).^38^ Parent reports will be used for ages 10 and 16, and self-reports will be used for age 25. In TEDS, irritability will be assessed using 1 item (i.e. ‘I get very angry and often lose my temper’) from the conduct problems subscale of SDQ.^36^ Parent reports will be used at ages 9 and 16, and self-reports at ages 21.

**Anxiety.** Two measures will be used to assess anxiety in ALSPAC. The mother-rated generalised anxiety (GAD) symptoms score from DAWBA^38^ will be used at age 10, while the self-reported measure will be used at age 16. At age 25, a self-reported Screen for Adult Anxiety Related Disorders (SCAARED)^39^ will be used. In TEDS, two measures will be used to assess anxiety – a parent-reported Anxiety-Related Behaviours Questionnaire (ARBQ)^40^ at ages 9 and 16 and a self-reported 10-item Generalised Anxiety Disorder Dimensional Scale (GAD-D)^41^ at age 21.

**Emotion recognition.** In ALSPAC, emotion recognition will be assessed using two facial emotion recognition tasks: the facial expression subtest from the Diagnostic Analysis of Non-Verbal Accuracy (DANVA)^42^ at age 8 and the Emotion Recognition Task (ERT)^43^ at age 24. During these tasks, individuals were presented with images of faces on the screen and asked to assign an emotion (e.g. happy, sad, angry, disgusted, surprised, and fearful). At age 8, the intensity of facial expressions was either low or high, while at age 24, there were 8 levels of intensity. We will use the global measure of incorrectly identified emotions by summing up emotion-specific misattributions across all emotions (i.e. by summing up the number of faces misattributed as happy, sad, angry, and fearful).

**Response inhibition.** The Stop-Signal Task^44^ will be used to assess response inhibition (i.e. impulse control) in ALSPAC at ages 11, 17 and 24. During the task, individuals were asked to press the corresponding button to the letter presented on a screen (X or O) as fast as they could but inhibit their response (i.e. not press the button) if they heard an auditory stop signal (i.e. a beep) after the appearance of a letter. The accuracy (i.e. the number of correct responses) on stop signal trials will be used as a primary measure, with a higher score indicating a better response inhibition (i.e. fewer errors).

**Working memory.** Two measures will be used to assess working memory capacity: the Counting Span Task (CST)^45^ at age 11 and the N-Back task (NBT)^46^ at ages 17 and 24. During the CST task, children were presented with the number of red and blue dots on the screen and asked to point to and count the number of red dots out loud (i.e. processing component). After each set, children were asked to recall the number of red dots on all previous trials in the order they were presented within that set (i.e. storage component). Analyses will use a child’s working memory span score: the number of correctly recalled sets weighted by the number of screens within each set automatically calculated by the computer program. During the NBT, participants had to monitor the screen where numbers (0 to 9) were presented, and they were asked to press the key ‘1’ if a stimulus (i.e. number) matched the one two trials before (i.e. 2-back design) or press the key ‘2’ if it did not. A primary measure will be a discriminability index (d’), which captures an individual’s ability to distinguish signal from noise, with a higher d’ indicating better working memory.

**Sustained attention.** Sustained attention will be assessed using two measures: the Test of Everyday Attention for Children (TEA-CH) Sky Search Dual Task (SSDT)^47^ at age 11, and the online version of Sustained Attention to Response Task (SART)^48^ at age 25. During the SSDT, children had to combine two tasks: circle pairs of identical spaceships as quickly as possible, avoid making errors by missing any sets of spaceships, and count the number of spaceship noises during the task. We will use a dual-task decrement score rather than a normative score, as ALSPAC documentation recommends. During the SART task, digits are presented on the computer screen, and participants are asked to respond by pressing a key to all digits except the pre-specified no-go digit that appears every nine digits.

**Locus of control (LoC).** In ALSPAC, LoC will be assessed using a self-reported shortened version of the Children’s Nowicki-Strickland Internal External Control Scale (CNSIE)^49^ at ages 8 and 16.

**Negative cognitive style.** In ALSPAC, negative cognitive style will be assessed using a self-reported Cognitive Styles Questionnaire Short Form (CSQ-SF)^50^ at age 17. Participants are presented with 8 negative hypothetical situations and asked if these are caused by internal vs external (i.e. themselves vs others), specific vs global (i.e. the implications are specific to this particular situation vs it will impact other areas of life), stable vs unstable (i.e. the degree to which the cause will persist and will lead to failures in the future) factors. Participants also had to rate the degree to which causes of negative events reflect their self-worth (i.e. that they are flawed).

**Statistical analysis**

All analyses will be performed in Stata version 17. Mediation (see **Figure 1**) will be performed using a counterfactual approach, enabling us to incorporate exposure-mediator interactions in our mediation analyses.^51^ We will use this approach to decompose the effect of ADHD on depression via direct effects and indirect effects through clinical, cognitive-affective, and negative thought patterns mediators. Specifically, we will estimate the pure natural direct effects (PNDE) (i.e. the effect of ADHD on depression when the mediator is set to the value it would take if the individual did not have ADHD), the total natural indirect effects (TNIE) (i.e. the effect of high levels of ADHD on depression that occurs because higher levels of the mediator are observed for those with high versus low ADHD scores), and proportion mediated.

**Primary analyses.** First, analyses will simultaneously include all potential mediators to avoid potential biases arising from examining them separately when mediators affect one another or if multiple mediators share an omitted common cause.^51^ We will, therefore, derive a single indirect effect from this model. Given the potential number of exposure-mediator interaction effects, we will examine each individually to assess whether they need to be included in the model. Interaction terms that will change the estimates of TNIE by more than half a standard error will be included in the models.^52^

Then, we will examine the relative contribution of each mediator category (i.e. cognitive-affective, negative thought patterns, and clinical) across development (i.e. childhood, adolescence, and young adulthood) by performing separate mediation models for each mediator category at each developmental stage. In these models, the other mediator categories will be treated as intermediate confounders in accordance with the DAG (**Figure 2**) (i.e. cognitive-affective mediators will be included as confounders in both the negative thought patterns and clinical models, and negative thought patterns will be included as confounders in the clinical models).

Finally, we will explore specific factors/hypothesised mediators (e.g. emotion recognition). Ideally, to determine the individual contribution of each factor/potential mediator without introducing bias, we would need to either assume that these factors are conditionally independent within the context of the specified model (i.e. there is no causal relationship between them) or specify unidirectional causal relationships between them. Since there is insufficient evidence to support these assumptions and previous research suggests that developmental differences tend to emerge in ADHD-mediator paths,^13,14,24,29^ we will focus on the exposure-mediator pathway as an indicator of how important the potential mediators are for young people with ADHD at different developmental periods. We will use regression models to examine whether associations between ADHD and hypothesised mediators differ across childhood, adolescence, and young adulthood, controlling for baseline confounders. Differences of ≥20% in proportion mediated or estimated effect sizes will be considered evidence for developmental differences (see **Table S1**, available online).

**Secondary analyses.** Since sex interactions cannot be examined in *g-formula* (i.e. it does not produce interaction parameters, only mediated effect accounting for the interaction), sex differences in the mediation models will be examined by stratifying analyses by sex. Differences of ≥20% in proportion mediated will be considered evidence for sex differences.^53^ In regression models, sex differences will be examined by testing ADHD and sex interactions.

**Confounders**. Counterfactual mediation sets out the assumptions required for estimating causal direct and indirect effects. It assumes 1) no unmeasured confounding (i.e. that all confounders are measured accurately and included in the model) in the exposure-outcome relationship; 2) no unmeasured confounding in the mediator-outcome relationship; 3) no unmeasured confounding in the exposure-mediator relationship; 4) absence of intermediate confounders: there are no mediator-outcome confounders that are affected by the exposure.^51^ Therefore, to inform the choice of which variables to include in a model to adjust for exposure-outcome, exposure-mediator, and mediator-outcome confounders, we developed a directed acyclic graph (DAG) (see **Figure 2**; for more detailed DAG, see **Figure S1**, available online). Analyses will be adjusted for a combination of potential baseline confounders (i.e. potential causes of ADHD, mediators and depression), intermediate confounders (i.e. potential outcomes of ADHD that are potential causes of mediators and depression) and mediator-outcome confounders (i.e. potential causes of mediators and depression). Baseline confounders, assessed prior to ADHD, will include maternal education/qualifications as an index of family socioeconomic status, maternal depression as an index of maternal mental health, and offspring sex and age. Intermediate confounders, assessed concurrently/after ADHD and prior to mediators, will include peer relationship quality for all models and parent hostility for childhood models, as this variable is unavailable in adolescence and young adulthood. Mediator-outcome confounders, assessed prior to the mediators and depression, will include pubertal status in the adolescence models only since these data are not available before childhood mediators are measured.

**Model Estimation.** Mediation analyses will be run using *g-formula* in Stata, which enables the inclusion of intermediate confounders (see **Figure 2**). In TEDS, analyses will cluster data by family ID, and robust maximum likelihood estimates will be used to obtain appropriate standard errors.

**Missing data.** We will seek to impute data for incomplete variables and study missing data patterns and predictors of missingness to guide the imputation process using The Treatment and Reporting of Missing Data in Observational Studies Framework.^54^ For more details on multiple imputation steps and procedures, see **Supplement 2**, available online.

**Timeline for completion of the study**

Analyses and writing will be completed within 9 months of pre-registration acceptance.

**Data Access Certification**

The authors have not had access to the full data. Some authors have had access to some of the ALSPAC and TEDS variables for other research papers.

| **Table 1.** Measures used in both cohorts. | | | | | | |
| --- | --- | --- | --- | --- | --- | --- |
| **Construct** | **Measure** | **Scoring** | **Items** | **Score range** | **Informant** | **Age** |
| **The Avon Longitudinal Study of Parents and Children** | | | | | | |
| ADHD | SDQ hyperactivity/inattention subscale | 3-point Likert (0-2) | 5 | 0-10 | Parent | 7, 13, 17 |
| Depression | sMFQ | 3-point Likert (0-2) | 13 | 0-26 | Self | 12, 18, 27 |
| Irritability | DAWBA ODD section | 3-point Likert (0-2) | 3 | 0-6 | Parent | 10, 16 |
|  |  |  |  |  | Self | 25 |
| Anxiety | DAWBA GAD section | 4-point Likert (0-3) | 7 | 0-21 | Parent | 10, 16 |
|  | SCAARED GAD subscale | 3-point Likert (0-2) | 13 | 0-26 | Self | 25 |
| Emotion recognition | DANVA facial expression subtest | Task | - | 0-24 | Self | 8 |
|  | ERT | Task | - | Continuous | Self | 24 |
| Response inhibition | SST | Task | - | Continuous | Self | 11, 17, 24 |
| Working memory | CST | Task | - | 0-5 | Self | 11 |
|  | NBT | Task | - | Continuous | Self | 17, 24 |
| Sustained attention | TEA-CH SSDT | Task | - | Continuous | Self | 11 |
|  | SART | Task | - | Continuous | Self | 25 |
| Locus of control | CNSIE | 2-point Likert (1-2) | 12 | 12-24 | Self | 8, 16 |
| Negative cognitive style | CSQ-SF | 5-point Likert (1-5) | 64 | 64-320 | Self | 17 |
| **The Twins Early Development Study (TEDS)** | | | | | | |
| ADHD | SDQ hyperactivity/inattention subscale | 3-point Likert (0-2) | 5 | 0-10 | Parent | 7, 12, 16 |
| Depression | sMFQ | 3-point Likert (0-2) | 13 | 0-26 | Self | 12, 21, 26 |
| Irritability | SDQ conduct subscale | 3-point Likert (0-2) | 1 | 0-2 | Parent | 9, 16 |
|  |  |  |  |  | Self | 21 |
| Anxiety | ARBQ | 3-point Likert (0-2) | 16 | 0-32 | Parent | 9, 16 |
|  | GAD-D | 5-point Likert (0-4) | 10 | 0-40 | Self | 21 |
| **Note.** ADHD = Attention Deficit Hyperactivity Disorder; SDQ = Strengths and Difficulties Questionnaire; sMFQ = short Mood and Feelings Questionnaire; DAWBA = Development and Well-Being Assessment; ODD = Oppositional Defiant Disorder; GAD = Generalised Anxiety Disorder; SCAARED = Screen for Adult Anxiety Related Disorders; DANVA = Diagnostic Analysis of Non-Verbal Accuracy; ERT = Emotion Recognition Task; SST = Stop-Signal Task; CST = Counting Span Task; NBT = N-Back task; TEA-CH = Test of Everyday Attention for Children; SSDT = Sky Search Dual Task; SART = Sustained Attention to Response Task; CNSIE = Children’s Nowicki-Strickland Internal External Control Scale; CSQ-SF = Cognitive Styles Questionnaire Short Form; ARBQ = Anxiety-Related Behaviours Questionnaire; GAD-D = Generalised Anxiety Disorder Dimensional Scale. | | | | | | |

**Figure 1: Time of assessments for ADHD, hypothesised mediators and depressive symptoms in childhood, adolescence, and young adulthood in both cohorts**

**Note:** ALSPAC = Avon Longitudinal Study of Parents and Children; TEDS = Twins Early Development Study; ADHD = Attention Deficit Hyperactivity Disorder; DAWBA = Development and Well-Being Assessment; DANVA = Diagnostic Analysis of Non-Verbal Accuracy; SST = Stop-Signal Task; CST = Counting Span Task; SSDT = Sky Search Dual Task; CNSIE = Children’s Nowicki-Strickland Internal External Control Scale; NBT = N-Back task; CSQ-SF = Cognitive Styles Questionnaire Short Form; SCAARED = Screen for Adult Anxiety Related Disorders; ERT = Emotion Recognition Task; SART = Sustained Attention to Response Task; SDQ = Strengths and Difficulties Questionnaire; sMFQ = short Mood and Feelings Questionnaire; ARBQ = Anxiety-Related Behaviours Questionnaire; GAD-D = Generalised Anxiety Disorder Dimensional Scale; y = years.

**Figure 2: Directed Acyclic Graph (DAG) describing potential measured and unmeasured confounders**

**Note:** DAG visualise variables of interest (i.e. nodes, measured or unmeasured) and causal relationships between them (i.e. arrows pointing in one direction, from cause to effect, representing ‘paths’). ADHD is an exposure, clinical and cognitive-affective mechanisms are mediators, and depression is an outcome. All arrows to/from the box represent arrows to all three categories of mediators within the box. Clinical mechanisms will comprise irritability and anxiety, cognitive-affective will comprise emotion recognition, response inhibition, working memory, and sustained attention, while negative thought patterns will comprise locus of control and negative cognitive style. To deal with confounding, analyses need to block the backdoor paths by conditioning (i.e. controlling for) the variables that are the common cause (i.e. confounder) of the two or more variables. Theoretically, analyses would need to be adjusted for baseline confounders, mediation-outcome confounders, and intermediate confounders. However, in this case, some of the confounders are unmeasured, such as genetic confounding and school transition. Available confounders to be included in the analyses are described in the analysis section. SES = socioeconomic status; MH = mental health; IQ = intelligence quotient; ACEs = adverse childhood experiences; ADHD = attention deficit hyperactivity disorder. The box in the middle simplifies the DAG and uses fewer arrows pointing toward individual mediators.

**References**

1. Salari N, Ghasemi H, Abdoli N, et al. The global prevalence of ADHD in children and adolescents: a systematic review and meta-analysis. *Ital J Pediatr*. 2023;49(1). doi:10.1186/S13052-023-01456-1

2. Ayano G, Tsegay L, Gizachew Y, et al. Prevalence of attention deficit hyperactivity disorder in adults: Umbrella review of evidence generated across the globe. *Psychiatry Res*. 2023;328:115449. doi:10.1016/j.psychres.2023.115449

3. Choi WS, Woo YS, Wang SM, Lim HK, Bahk WM. The prevalence of psychiatric comorbidities in adult ADHD compared with non-ADHD populations: A systematic literature review. *PLoS One*. 2022;17(11):e0277175. doi:10.1371/journal.pone.0277175

4. Angold A, Costello EJ, Erkanli A. Comorbidity. *J Child Psychol Psychiat*. 1999;40(1):57-87. doi:10.1111/1469-7610.00424

5. Chronis-Tuscano A, Molina BSG, Pelham WE, et al. Very Early Predictors of Adolescent Depression and Suicide Attempts in Children With Attention-Deficit/Hyperactivity Disorder. *Arch Gen Psychiatry*. 2010;67(10).

6. Meinzer MC, Lewinsohn PM, Pettit JW, et al. Attention-deficit/hyperactivity disorder in adolescence predicts onset of major depressive disorder through early adulthood. *Depression and Anxiety* . 2013;30:546-553. doi:10.1002/da.22082

7. Biederman J, Ball SW, Monuteaux MC, et al. New insights into the comorbidity between ADHD and major depression in adolescent and young adult females. *J Am Acad Child Adolesc Psychiatry*. 2008;47(4):426-434. doi:10.1097/CHI.0B013E31816429D3

8. Garcia-Argibay M, Brikell I, Thapar A, et al. Attention-Deficit/Hyperactivity Disorder and Major Depressive Disorder: Evidence From Multiple Genetically Informed Designs. *Biol Psychiatry*. 2024;95(5):444-452. doi:10.1016/J.BIOPSYCH.2023.07.017

9. Thapar A, Livingston LA, Eyre O, Riglin L. Practitioner Review: Attention-deficit hyperactivity disorder and autism spectrum disorder – the importance of depression. *J Child Psychol Psychiatry*. 2023;64(1):4-15. doi:10.1111/JCPP.13678

10. Guo C, Assumpcao L, Hu Z. Efficacy of Non-pharmacological Treatments on Emotional Symptoms of Children and Adults with Attention-Deficit/Hyperactivity Disorder: A Meta-Analysis. *J Atten Disord*. 2022;26(4):508-524. doi:10.1177/10870547211001953

11. Rice F, Sellers R, Hammerton G, et al. Antecedents of New-Onset Major Depressive Disorder in Children and Adolescents at High Familial Risk. *JAMA Psychiatry*. 2017;74(2):153-160. doi:10.1001/JAMAPSYCHIATRY.2016.3140

12. Quenneville AF, Kalogeropoulou E, Nicastro R, Weibel S, Chanut F, Perroud N. Anxiety disorders in adult ADHD: A frequent comorbidity and a risk factor for externalizing problems. *Psychiatry Res*. 2022;310:114423. doi:10.1016/J.PSYCHRES.2022.114423

13. D’Agati E, Curatolo P, Mazzone L. Comorbidity between ADHD and anxiety disorders across the lifespan. *Int J Psychiatry Clin Pract*. 2019;23(4):238-244. doi:10.1080/13651501.2019.1628277

14. Shaw P, Stringaris A, Nigg J, Leibenluft E. Emotion dysregulation in attention deficit hyperactivity disorder. *Am J Psychiatry*. 2014;171(3):276-293. doi:10.1176/APPI.AJP.2013.13070966

15. Eyre O, Hughes RA, Thapar AK, et al. Childhood neurodevelopmental difficulties and risk of adolescent depression: the role of irritability. *Journal of Child Psychology and Psychiatry*. 2019;60(8):866-874. doi:10.1111/JCPP.13053

16. Roy A, Oldehinkel AJ, Verhulst FC, Ormel J, Hartman CA. Anxiety and Disruptive Behavior Mediate Pathways From Attention-Deficit/Hyperactivity Disorder to Depression. *J Clin Psychiatry*. 2014;75(2):1189. doi:10.4088/JCP.13M08648

17. Willcutt EG, Doyle AE, Nigg JT, Faraone S V., Pennington BF. Validity of the executive function theory of attention-deficit/hyperactivity disorder: a meta-analytic review. *Biol Psychiatry*. 2005;57(11):1336-1346. doi:10.1016/J.BIOPSYCH.2005.02.006

18. Agha SS, Riglin L, Carbury R, et al. Young Adult ADHD Symptoms in the General Population and Neurocognitive Impairment. *J Atten Disord*. 2024;28(1):89-98. doi:10.1177/10870547231201870

19. Rock PL, Roiser JP, Riedel WJ, Blackwell AD. Cognitive impairment in depression: a systematic review and meta-analysis. *Psychol Med*. 2014;44(10):2029-2040. doi:10.1017/S0033291713002535

20. Joormann J, Gotlib IH. Emotion regulation in depression: Relation to cognitive inhibition. *Cogn Emot*. 2010;24(2):281-298. doi:10.1177/0963721410370293

21. Mayer JS, Bernhard A, Fann N, et al. Cognitive mechanisms underlying depressive disorders in ADHD: A systematic review. *Neurosci Biobehav Rev*. 2021;121:307-345. doi:10.1016/J.NEUBIOREV.2020.12.018

22. Broletti MC, Efthymiou C, Murray AL, McDougal E, Rhodes SM. Investigating the Mediating Role of Executive Function in the Relationship Between ADHD and DCD Symptoms and Depression in Adults. *J Autism Dev Disord*. Published online November 15, 2023:1-13. doi:10.1007/S10803-023-06148-7/TABLES/8

23. Haza B, Gosling CJ, Ciminaghi F, Conty L, Pinabiaux C. Research Review: Social cognition and everyday social skills in children and adolescents with attention‐deficit/hyperactivity disorder: a meta‐analysis of case–control studies. *Journal of Child Psychology and Psychiatry*. Published online June 11, 2024. doi:10.1111/jcpp.14006

24. Olaya-Galindo MD, Vargas-Cifuentes OA, Vélez Van-Meerbeke A, Talero-Gutiérrez C. Establishing the Relationship Between Attention Deficit Hyperactivity Disorder and Emotional Facial Expression Recognition Deficit: A Systematic Review. *J Atten Disord*. 2023;27(11):1181-1195. doi:10.1177/10870547231154901

25. Dalili MN, Penton-Voak IS, Harmer CJ, Munafò MR. Meta-analysis of emotion recognition deficits in major depressive disorder. *Psychol Med*. 2015;45(6):1135-1144. doi:10.1017/S0033291714002591

26. Beck AT. *Cognitive Therapy and the Emotional Disorders*. Penguin; 1979.

27. Abramson LY, Metalsky GI, Alloy LB. Hopelessness Depression: A Theory-Based Subtype of Depression. *Psychol Rev*. 1989;96(2):358-372. doi:10.1037/0033-295X.96.2.358

28. Rucklidge J, Brown D, Crawford S, Kaplan B. Attributional Styles and Psychosocial Functioning of Adults With ADHD. *http://dx.doi.org/101177/1087054706289942*. 2007;10(3):288-298. doi:10.1177/1087054706289942

29. Ostrander R, Herman KC. Potential cognitive, parenting, and developmental mediators of the relationship between ADHD and depression. *J Consult Clin Psychol*. 2006;74(1):89-98. doi:10.1037/0022-006X.74.1.89

30. Knouse LE, Zvorsky I, Safren SA. Depression in Adults with Attention-Deficit/Hyperactivity Disorder (ADHD): The mediating role of cognitive-behavioral factors. *Cognit Ther Res*. 2013;37(6):1220-1232. doi:10.1007/S10608-013-9569-5

31. Kendler KS, Gardner CO. Sex differences in the pathways to major depression: a study of opposite-sex twin pairs. *Am J Psychiatry*. 2014;171(4):426-435. doi:10.1176/APPI.AJP.2013.13101375

32. Boyd A, Golding J, Macleod J, et al. Cohort profile: The ’Children of the 90s’-The index offspring of the avon longitudinal study of parents and children. *Int J Epidemiol*. 2013;42(1):111-127. doi:10.1093/IJE/DYS064

33. Fraser A, Macdonald-wallis C, Tilling K, et al. Cohort Profile: the Avon Longitudinal Study of Parents and Children: ALSPAC mothers cohort. *Int J Epidemiol*. 2013;42(1):97-110. doi:10.1093/IJE/DYS066

34. Lockhart C, Bright J, Ahmadzadeh Y, et al. Twins Early Development Study (TEDS): A genetically sensitive investigation of mental health outcomes in the mid-twenties. *JCPP Advances*. 2023;3(2):e12154. doi:10.1002/JCV2.12154

35. Rimfeld K, Malanchini M, Spargo T, et al. Twins Early Development Study: A Genetically Sensitive Investigation into Behavioral and Cognitive Development from Infancy to Emerging Adulthood. *Twin Research and Human Genetics*. 2019;22(6):508-513. doi:10.1017/THG.2019.56

36. Goodman R. The Strengths and Difficulties Questionnaire: a research note. *J Child Psychol Psychiatry*. 1997;38(5):581-586. doi:10.1111/J.1469-7610.1997.TB01545.X

37. Messer SC, Angold A, Costello EJ, Loeber R, Van Kammen W, Stouthamer-Loeber M. Development of a short questionnaire for use in epidemiological studies of depression in children and adolescents: Factor composition and structure across development. 1995;5:25-262.

38. Goodman R, Ford T, Richards H, Gatward R, Meltzer H. The Development and Well‐Being Assessment: Description and Initial Validation of an Integrated Assessment of Child and Adolescent Psychopathology. *Journal of Child Psychology and Psychiatry*. 2000;41(5):645-655. doi:10.1111/J.1469-7610.2000.TB02345.X

39. Angulo M, Rooks BT, Gill MK, et al. Psychometrics of the Screen for Adult Anxiety Related Disorders (SCAARED)- A New Scale for the Assessment of DSM-5 Anxiety Disorders. *Psychiatry Res*. 2017;253:84. doi:10.1016/J.PSYCHRES.2017.02.034

40. Eley TC, Bolton D, O’Connor TG, Perrin S, Smith P, Plomin R. A twin study of anxiety-related behaviours in pre-school children. *J Child Psychol Psychiatry*. 2003;44(7):945-960. doi:10.1111/1469-7610.00179

41. Lebeau RT, Glenn DE, Hanover LN, Beesdo‐Baum K, Wittchen H, Craske MG. A dimensional approach to measuring anxiety for DSM‐5. *Int J Methods Psychiatr Res*. 2012;21(4):258-272. doi:10.1002/mpr.1369

42. Nowicki S, Duke MP. Individual differences in the nonverbal communication of affect: The diagnostic analysis of nonverbal accuracy scale. *J Nonverbal Behav*. 1994;18(1):9-35. doi:10.1007/BF02169077/METRICS

43. Penton-Voak IS, Bate H, Lewis G, Munafò MR. Effects of emotion perception training on mood in undergraduate students: randomised controlled trial. *The British Journal of Psychiatry*. 2012;201:71-72. doi:10.1192/bjp.bp.111.107086

44. Logan GD, Cowan WB. On the ability to inhibit thought and action: A theory of an act of control. *Psychol Rev*. 1984;91(3):295-327. doi:10.1037/0033-295X.91.3.295

45. Case R, Kurland DM, Goldberg J. Operational efficiency and the growth of short-term memory span. *J Exp Child Psychol*. 1982;33(3):386-404. doi:10.1016/0022-0965(82)90054-6

46. Kirchner WK. Age differences in short-term retention of rapidly changing information. *J Exp Psychol*. 1958;55(4):352-358. doi:10.1037/H0043688

47. Manly T, Anderson V, Nimmo-Smith I, Turner A, Watson P, Robertson IH. The differential assessment of children’s attention: the Test of Everyday Attention for Children (TEA-Ch), normative sample and ADHD performance. *J Child Psychol Psychiatry*. 2001;42(8):1065-1081. doi:10.1111/1469-7610.00806

48. Bellgrove MA, Hawi Z, Lowe N, Kirley A, Robertson IH, Gill M. DRD4 gene variants and sustained attention in attention deficit hyperactivity disorder (ADHD): Effects of associated alleles at the VNTR and −521 SNP. *American Journal of Medical Genetics Part B: Neuropsychiatric Genetics*. 2005;136B(1):81-86. doi:10.1002/AJMG.B.30193

49. Nowicki S, Strickland BR. A locus of control scale for children. *J Consult Clin Psychol*. 1973;40(1):148-154. doi:10.1037/H0033978

50. Meins E, McCarthy-Jones S, Fernyhough C, Lewis G, Bentall RP, Alloy LB. Assessing negative cognitive style: Development and validation of a Short-Form version of the Cognitive Style Questionnaire. *Pers Individ Dif*. 2012;52(5-2):581. doi:10.1016/J.PAID.2011.11.026

51. Van Der Weele T, Vansteelandt S. Mediation analysis with multiple mediators. *Epidemiol Methods*. 2013;2(1):95-115. doi:10.1515/EM-2012-0010/ASSET/GRAPHIC/EM-2012-0010_INLINE75.PNG

52. Vanderweele TJ. *Explanation in Causal Inference: Methods for Mediation and Interaction.* Oxford University Press; 2015.

53. Cohen J. *Statistical Power Analysis for the Behavioral Sciences*. Routledge; 2013. doi:10.4324/9780203771587

54. Lee KJ, Tilling KM, Cornish RP, et al. Framework for the treatment and reporting of missing data in observational studies: The Treatment And Reporting of Missing data in Observational Studies framework. *J Clin Epidemiol*. 2021;134:79-88. doi:10.1016/j.jclinepi.2021.01.008
