# Supplementary material for: Study Preregistration: Clinical and Cognitive Mediators Underlying Subsequent Depression in Individuals With Attention-Deficit/Hyperactivity Disorder: A Developmental Approach
Source: J Am Acad Child Adolesc Psychiatry. 2025 Nov;64(11):1329–31. doi: 10.1016/j.jaac.2025.03.023 (PMC13187293; doi:10.1016/j.jaac.2025.03.023)
Supplement: Supplementary Material [file mmc2.docx]

**Supplement 1. The Avon Longitudinal Study of Parents and Children (ALSPAC)**

**Cohort description**

Pregnant women resident in Avon, UK, with expected dates of delivery between 1st April 1991 and 31st December 1992, were invited to take part in the study. 20,248 pregnancies have been identified as being eligible, and the initial number of pregnancies enrolled was 14,541. Of the initial pregnancies, there was a total of 14,676 foetuses, resulting in 14,062 live births and 13,988 children who were alive at 1 year of age. When the oldest children were approximately 7 years of age, an attempt was made to bolster the initial sample with eligible cases who had failed to join the study originally. As a result, when considering variables collected from the age of seven onwards (and potentially abstracted from obstetric notes) there are data available for more than the 14,541 pregnancies mentioned above: The number of new pregnancies not in the initial sample (known as Phase I enrolment) that are currently represented in the released data and reflecting enrolment status at the age of 24 is 906, resulting in an additional 913 children being enrolled (456, 262 and 195 recruited during Phases II, III and IV respectively). The total sample size for analyses using any data collected after the age of seven is therefore 15,447 pregnancies, resulting in 15,658 foetuses. Of these 14,901 children were alive at 1 year of age. Of the original 14,541 initial pregnancies, 338 were from a woman who had already enrolled with a previous pregnancy, meaning 14,203 unique mothers were initially enrolled in the study. As a result of the additional phases of recruitment, a further 630 women who did not enrol originally have provided data since their child was 7 years of age. This provides a total of 14,833 unique women (G0 mothers) enrolled in ALSPAC as of September 2021. G0 partners were invited to complete questionnaires by the mothers at the start of the study and they were not formally enrolled at that time. 12,113 G0 partners have been in contact with the study by providing data and/or formally enrolling when this started in 2010. 3,807 G0 partners are currently enrolled.

Data were collected and managed using The Research Electronic Data Capture (REDCap).^1,2^ Ethical approval for the study was obtained from the ALSPAC Law and Ethics and Local Research Ethics Committees. Informed consent for the use of data collected via questionnaires and clinics was obtained from participants following the recommendations of the ALSPAC Ethics and Law Committee at the time. Further details on the sample and measures can be found elsewhere.^3–6^ Please note that the study website contains details of all the data that is available through a fully searchable data dictionary and variable search tool: http://www.bristol.ac.uk/alspac/researchers/our-data/.

| **Table S1.** The study aims and planned analyses. | | |
| --- | --- | --- |
| **Aim** | **Analysis** | **Output/ decision criteria** |
| **Aim 1:** The mediating role of cognitive-affective, negative thought, and clinical mediators | Counterfactual mediation with all mediators included simultaneously    3 models in total (childhood, adolescence and young adulthood) | Proportion mediated (%) of the total effect through all potential clinical and cognitive mediators    Further analyses will be performed if the proportion mediated will be ≥10% |
| **Aim 2:** Individual contribution of three categories of mediators (i.e. cognitive-affective, negative thought processes, and clinical) | Counterfactual mediation with individual contributions of each mediator category    9 models in total (3 categories of mediators x 3 developmental) | Proportion mediated (%) from three separate mediation models (i.e. cognitive-affective, negative thought processes, and clinical)    Mediators with a proportion mediated ≥10% will be considered supported  Differences of ≥20% in proportion mediated will be considered evidence for developmental differences |
| **Aim 3:** Individual contribution of specific mediators/mechanisms | Regression models at each developmental stage for ADHD and potential mediator association    24 models in total (8 mediators x 3 developmental stages) | Standardised β coefficients for associations between ADHD and hypothesised mediators in childhood, adolescence, and young adulthood    Differences of ≥20% in estimated effect sizes will be considered evidence for developmental differences |
| **Secondary Aim:** Potential sex differences in all models | All analyses will be performed stratified by sex  72 models in total (3 aim 1 models, 9 aim 2 models, and 24 aim 3 models, all separately for males and females) | Proportion mediated (%) or standardised β coefficients, depending on the research question    Differences of ≥20% in proportion mediated or estimated effect sizes will be considered evidence for sex differences |
| **Note.** PA = primary aim; SA = secondary aim. All analyses will be adjusted for baseline confounders (i.e. maternal education/qualifications, maternal depression, offspring age and sex). Adolescent models will additionally be adjusted for mediator-outcome confounder pubertal status. Analyses will also account for intermediate confounders: maternal hostility and peer relationship quality in childhood models, and peer relationship quality in adolescence and young adulthood models. | | |


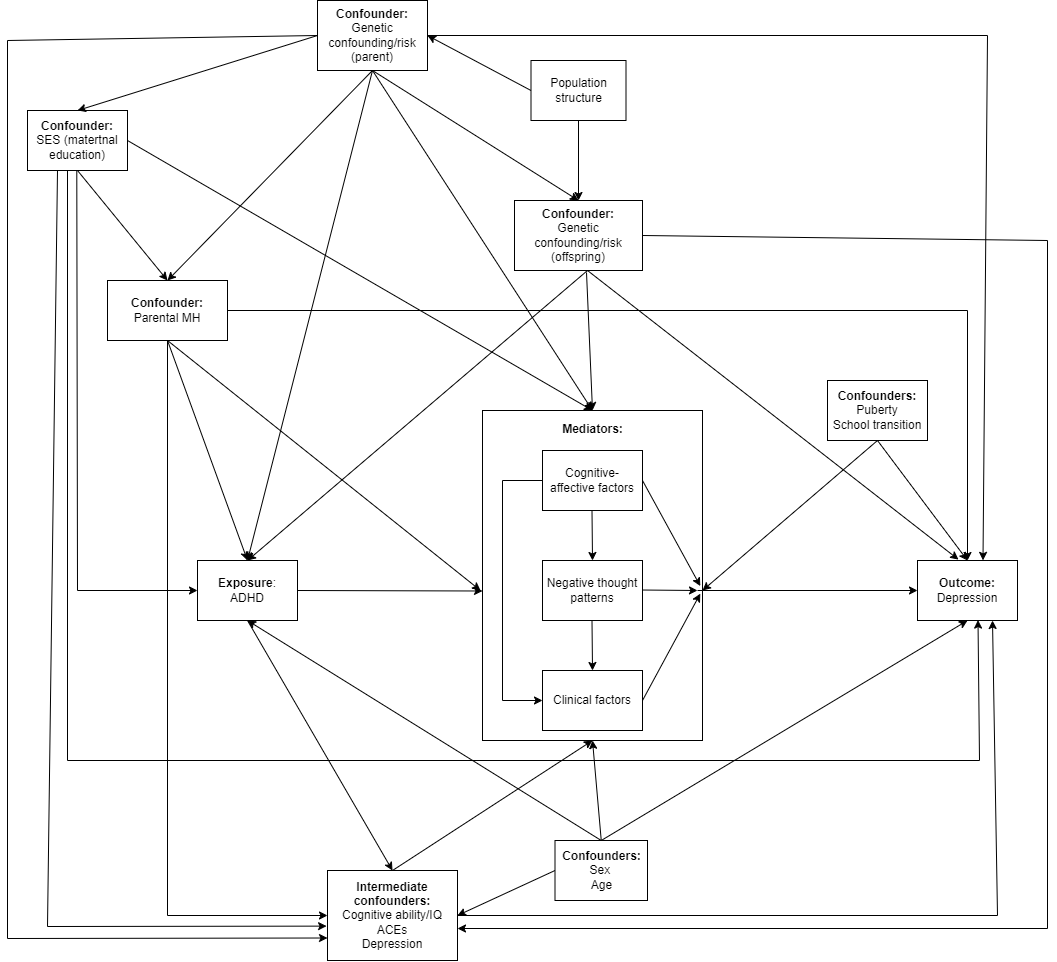


**Figure S1.** Detailed Directed Acyclic Graph (DAG)

**Supplement 2. Multiple Imputation**

First, we will investigate the missing data patterns and percentage of missingness in all variables of interest. Multiple imputation (MI) will be performed for all variables with partially missing data.^7^ We will not have a maximum threshold for the amount of missing data allowed since research suggests that MI reduces bias and might be beneficial even when the proportion of missing data is large.^8^ Multiple imputation assumes that data is missing at random (MAR) – differences in missing and observed variables can be entirely explained by other variables observed in the sample.^7^ Therefore, to make this assumption more plausible, we will identify predictors of missingness for use as auxiliary variables (i.e. additional variables associated with the variable being imputed that are often used in MI to reduce bias and improve the precision of the estimates) in the imputation models. We will perform multiple imputation using chained equations (MICE) using 10 regression-switching cycles and will generate 50 imputed datasets that will be combined using Rubin’s rules.^9^ We will impute 50 datasets and use the convergence plots and Monte Carlo errors to ensure that the number is sufficient. If not, we will impute 10 more datasets each time until the number is adequate. Sensitivity analyses will be performed to compare results in imputed and non-imputed datasets, although imputed data will be presented as primary results.

**References**

1. Harris PA, Taylor R, Thielke R, Payne J, Gonzalez N, Conde JG. Research electronic data capture (REDCap)—A metadata-driven methodology and workflow process for providing translational research informatics support. *J Biomed Inform*. 2009;42(2):377-381. doi:10.1016/J.JBI.2008.08.010

2. Harris PA, Taylor R, Minor BL, et al. The REDCap consortium: Building an international community of software platform partners. *J Biomed Inform*. 2019;95. doi:10.1016/J.JBI.2019.103208

3. Boyd A, Golding J, Macleod J, et al. Cohort profile: The ’Children of the 90s’-The index offspring of the avon longitudinal study of parents and children. *Int J Epidemiol*. 2013;42(1):111-127. doi:10.1093/IJE/DYS064

4. Fraser A, Macdonald-wallis C, Tilling K, et al. Cohort Profile: the Avon Longitudinal Study of Parents and Children: ALSPAC mothers cohort. *Int J Epidemiol*. 2013;42(1):97-110. doi:10.1093/IJE/DYS066

5. Northstone K, Lewcock M, Groom A, et al. The Avon Longitudinal Study of Parents and Children (ALSPAC): an update on the enrolled sample of index children in 2019. *Wellcome Open Res*. 2019;4. doi:10.12688/WELLCOMEOPENRES.15132.1

6. Major-Smith D, Heron J, Fraser A, Lawlor DA, Golding J, Northstone K. The Avon Longitudinal Study of Parents and Children (ALSPAC): a 2022 update on the enrolled sample of mothers and the associated baseline data. *Wellcome Open Res*. 2022;7:283. doi:10.12688/wellcomeopenres.18564.1

7. Lee KJ, Tilling KM, Cornish RP, et al. Framework for the treatment and reporting of missing data in observational studies: The Treatment And Reporting of Missing data in Observational Studies framework. *J Clin Epidemiol*. 2021;134:79-88. doi:10.1016/j.jclinepi.2021.01.008

8. Madley-Dowd P, Hughes R, Tilling K, Heron J. The proportion of missing data should not be used to guide decisions on multiple imputation. *J Clin Epidemiol*. 2019;110:63-73. doi:10.1016/j.jclinepi.2019.02.016

9. White IR, Royston P, Wood AM. Multiple imputation using chained equations: Issues and guidance for practice. *Stat Med*. 2011;30(4):377-399. doi:10.1002/sim.4067
